# Highly conductive colloidal carbon based suspension for flow-assisted electrochemical systems

**DOI:** 10.1016/j.isci.2021.102456

**Published:** 2021-04-20

**Authors:** Marco S. Alfonso, Hélène Parant, Jinkai Yuan, Wilfrid Neri, Eric Laurichesse, Katerina Kampioti, Annie Colin, Philippe Poulin

**Affiliations:** 1 Centre de Recherche Paul Pascal, CNRS, Université de Bordeaux, 115 Avenue Schweitzer, 33600, Pessac, France; 2 Université PSL, MIE-CBI ESPCI Paris 10 Rue Vauquelin, Paris 75005, France

**Keywords:** Electrochemical energy storage, Energy storage, Physical chemistry

## Abstract

Carbon suspension electrodes are promising for flow-assisted electrochemical energy storage systems. They serve as flowable electrodes in electrolyte solutions of flow batteries, or flow capacitors. They can also be used for other applications such as capacitive deionization of water. However, developments of such suspensions remain challenging. The suspensions should combine low viscosity and high electronic conductivity for optimized performances. In this work, we report a flowable aqueous carbon dispersion which exhibits a viscosity of only 2 Pa.s at a shear rate of 5 s^−1^ for a concentration of particles of 7 wt%. This suspension displays an electronic conductivity of 65 mS/cm, nearly two orders of magnitude greater than previously investigated related materials. The investigated suspensions are stabilized by sodium alginate and arabic gum in the presence of ammonium sulfate. Their use in flowable systems for the storage and discharge of electrical charges is demonstrated.

## Highlights

• Fluid and highly conductive carbon-based suspensions are reported
• The suspensions are potentially useful for flow-assisted electrochemical applications
• Charge and discharge of flowable electrodes is quantitatively demonstrated

## Graphical abstract

**Figure:**
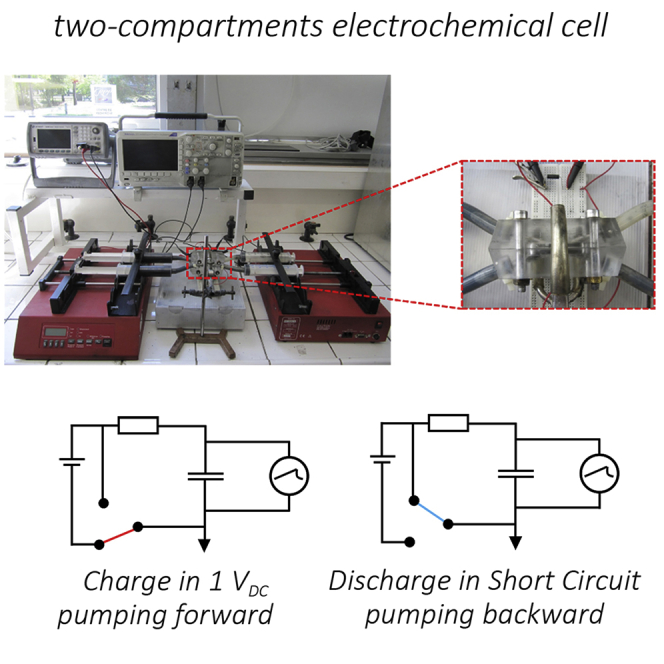

## Introduction

Extensive research is undertaken for the management of energy with new technologies that exploit renewable sources (Aneke and Wang, 2016; Akinyele and Rayudu, 2014; Chakrabarti et al., 2020). In the last decade, carbon black dispersions have been used as active material in different flow-assisted electrochemical energy storage systems (FAESs) (Skyllas-Kazacos et al., 2011; Skyllas Kazacos et al., 1986; Weber et al., 2011; Liu et al., 2020; Choi et al., 2020). In these systems, the carbon black dispersions serve as flowable electrodes in semi-solid flow batteries dispersed in an electrolyte solution (Duduta et al., 2011) or as active materials in the electrochemical flow capacitor (Presser et al., 2012) or in the flow capacitive deionization of water (Hatzell et al., 2014; Rommerskirchen et al., 2020). The major challenges toward efficient applications include the optimization of the rheological and electrical performances of flowable active material, namely, new classes of fluids that exhibit low viscosity and high electronic conductivity. Several studies proposed carbon black dispersions as good candidates for flowable electrodes. For instance, Li et al. (Li et al., 2013) have reported a conductivity value of 5 mS/cm for a dispersion of commercial Ketjenblack 0.2 wt% used in semi-solid flow cell. Subsequently, higher loadings of carbon filler were studied to achieve higher conductivities. For example, Paroda et al. (Porada et al., 2014) with a conductivity of 14 mS/cm, Dannison et al. (Dennison et al., 2014) and Hatzell et al. (Hatzell et al., 2017) with conductivities of 0.01 mS/cm and 0.3 mS/cm, and more recently Parant et al. (Parant et al., 2017) with a conductivity of a few mS/cm. In this latter work, the authors found an optimal formulation with commercial acetylene carbon black at a concentration of 8.0 wt% in water. They have also tested other carbon black materials but with less success because these other materials become too viscous with increasing concentration.

Indeed, in addition to electronic conductivity, viscosity is another critical property of carbon-based flowable electrodes. A low viscosity minimizes the energy needed for pumping and flowing the active fluids. Paroda et al. (Porada et al., 2014), have reported a viscosity equal to 8 Pa.s for a carbon concentration of 15 wt%, while Presser et al. (Presser et al., 2012) reported a viscosity of only 2 Pa.s for a concentration of 10 wt% of highly monodispersed carbon beads. Hatzell et al. (Hatzell et al., 2017) have reported a viscosity value of 10 Pa.s for a concentration of 20 wt%. Campos et al. (Campos et al., 2013) reported flowable carbon dispersions with a viscosity of 12 Pa.s for a concentration of 23 wt% of carbon material. Parant et al. (Parant et al., 2017) reported a viscosity above 30 Pa.s at a shear rate of 5 s^−1^ for optimal acetylene black materials. These results show that achieving a compromise of high conduction and flowability remains challenging (Madec et al., 2015; Akuzum et al., 2017, 2020; Boota et al., 2014; Hauptman et al., 2011; Helal et al., 2016).

The above state of the art is summarized in Table 1. Direct comparisons between these results and the ones presented in this work are not straightforward because of the different methodologies and materials presented in the literature.

**Table 1:** Examples of conductivity and viscosity from literature on colloidal carbon-based suspensions

Reference	Concentration (wt%)	Conductivity σ (mS/cm)	Viscosity η (Pa.s) @ 5 s^−1^
Presser et al. (2012)	10	Not reported	2
Li et al. (2013)	0.2	5	1.5
Paroda et al. (2014)	15	14	8
Dennison et al. (2014)	16	0.01	Not reported
Hatzell et al. (2014)	20	0.3	10
Parant et al. (2017)	8	4	30
Campos et al. (2013)	23	Not reported	11

Nevertheless, the above data provide guidance for ranges of conductivity and viscosity to be improved to progress further toward actual applications of flowable carbon dispersions.

We study in the present work dispersions reminiscent of the systems investigated by Parant et al. (Parant et al., 2017). We use indeed arabic gum as surfactant, and sodium alginate as polymeric stabilizer. The choice of arabic gum is due to its high dispersion ability, while sodium alginate prevents sedimentation of carbon particles over time as it increases the viscosity of the solution. Beyond these similarities, we present here a different formulation process, which includes the grinding of the carbon powder and the addition of a strong excess of ammonium sulfate and the use of Ketjenblack carbon. These modifications allow the viscosity to be kept at a low level and the electronic conductivity to be substantially increased up to 65 mS/cm, almost two orders of magnitude greater when compared to (Parant et al., 2017) for a concentration of carbon black of 7.0 wt%. The viscosity for this dispersion is of only 2 Pa.s, a value well below many of the above mentioned dispersions. We study the properties of these systems under flow, and show their potential application to store, and release charges under flow.

## Results and discussion

### Microstructure morphologies

Carbon black dispersions were deposited between glass slides for optical imaging by using an optical microscope (Leica DM 2500P) at room temperature. Figure S2 shows optical micrographs of Ketjenblack dispersions at different concentrations. It is possible to observe the agglomeration of the carbon black particles in large clusters with an average diameter of 5 μm. The size of the clusters grow with the concentration of particles. But it is difficult to confirm any percolation behavior in these conditions of confinement. Electrical measurements are preferentially used for this purpose.

### Rheological properties

**Figure 1:**
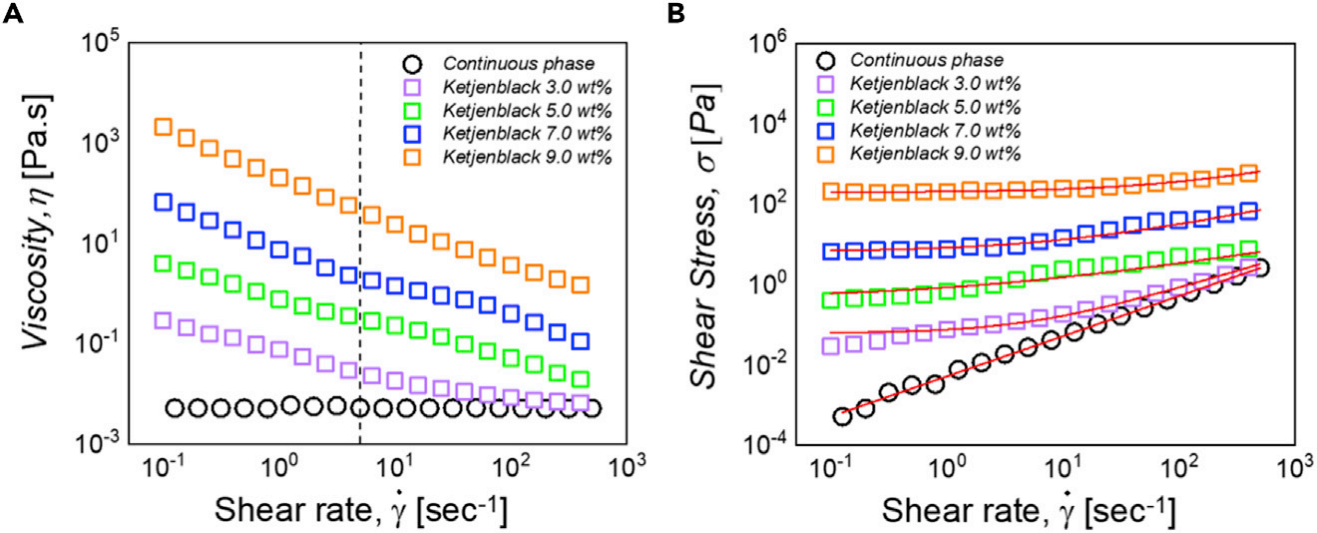
Rheology measurements of the Ketjenblack aqueous dispersion composed of 1.5 wt% arabic gum, 0.5 wt% sodium alginate and 2 M (NH_4_)_2_SO_4_ Several percentages of carbon are presented. (A) Viscosity of dispersions in Pa.s versus shear rate. (B) Shear stress of dispersions versus shear rate. Shear stress curves have been fitted using the Herschel-Bulkley law (red lines).

The rheological behaviors of the samples are presented in Figure 1, where the shear stress and viscosity are plotted as a function of the shear rate from 0.1 to 500 s^−1^.

We recall that the gap between the two plates was chosen so that the measurements are independent of its value. In this situation, wall slip can be considered as negligible (Yoshimura and Prud'homme, 1988).

The continuous phase exhibits a Newtonian viscosity of 0.05 Pa.s. It is observed that this viscosity is slightly lower than that reported by Parant et al. (Parant et al., 2017). The difference can be attributed to the presence of the electrolyte in strong excess which alters the rheological behavior of the continuous phase. It has been shown in particular that an increase in ionic strength can induce a decrease of the alginate persistence length associated to a decrease of viscosity (Zhang et al., 2001).

The addition of carbon particles induces the appearance of a yield stress, and the final dispersions show a shear thinning behavior with viscosity values lower than 100 Pa.s at low shear rate, for carbon black concentrations up to 7.0 wt%. A Herschel-Bulkley model for yield stress fluids can be applied to fit the rheology curves using the following equation:

(Equation 1) $$\sigma_{\left(z\right)}=\;\sigma_{0}\;+k\dot{\gamma \;}{\left(z\right)}^{n}$$

where, *σ*_(*z*)_ is shear stress, *σ*_0_ the yield stress, *γ*˙(z) the shear rate, *k* the consistency index, and *n* the flow index. Even for a small addition of carbon black particles, the final suspensions display a yield stress and a shear thinning behavior. These results suggest the formation of a network by the dispersed particles. The fitting parameters obtained by the Herschel-Bulkley model are given in Table 2 for each carbon dispersion. The viscosity measured at 5 s^−1^ is also shown in the table.

**Table 2:** Viscosity values in Pa.s measured at 5 s^−1^ and Herschel-Bulkley parameters deduced from the fit of the rheological data obtained in plate plate geometry

Parameter	Continuous Phase	Ketjenblack 3.0 wt%	Ketjenblack 5.0 wt%	Ketjenblack 7.0 wt%	Ketjenblack 9.0 wt%
*η @ 5 s*^*−1*^	0.005	0.02	0.3	2	45
*σ*_0_	0	0.059	0.74	7.18	194.50
*K*	0.005	0.14	0.17	1.25	8.56
*N*	1.0000	0.86	0.71	0.68	0.67

### Electronic conductivity

**Figure 2:**
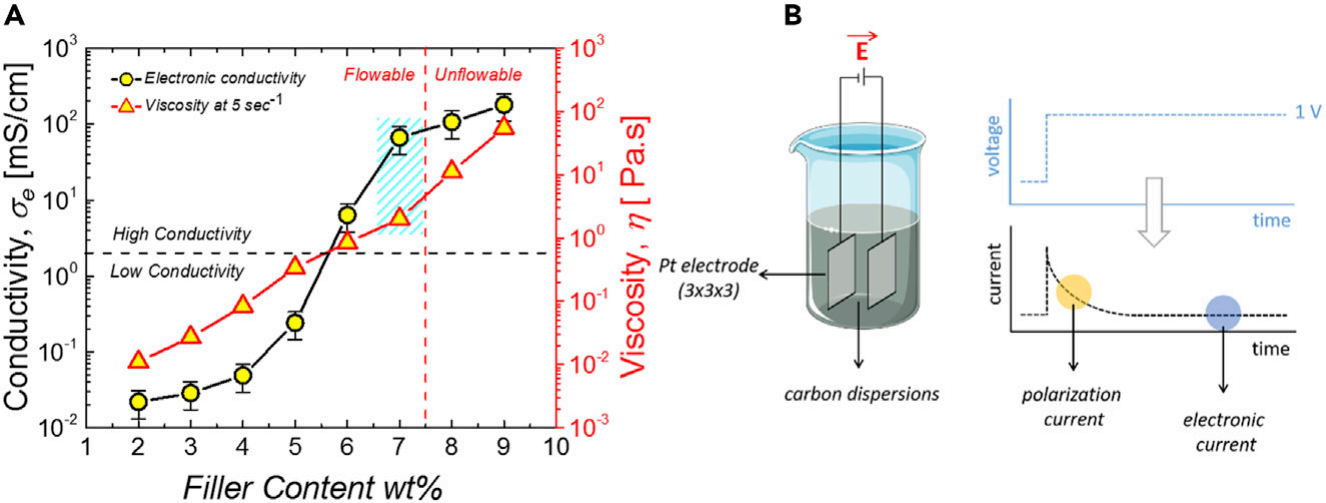
Electronic conductivity of the Ketjenblack aqueous dispersion composed of 1.5 wt% arabic gum, 0.5 wt% sodium alginate and 2 M (NH4)2SO4 (A) Electronic conductivity of dispersion of Ketjenblack in arabic gum 1.5 t% and sodium alginate 0.5 wt% in 2 M (NH_4_)_2_SO_4_ as a function of the filler content of carbon (wt%). (Error bars correspond to the standard deviation of 3 measurements). (B) DC chronoamperometry measurements of conductivity of the carbon dispersions. The current values related to the electronic conductivity are the stabilized values at the plateau. A square voltage of ±1 V is applied to the electrode for 2000 s in order to avoid the polarization currents due to the presence of free ions in the system.

Ketjenblack carbon dispersions are prepared at several filler weight percentages: from 2 wt% to 9 wt%. Figure 2 shows the evolution of the electronic conductivity of the carbon dispersions as a function of the filler content. At low carbon content, the dispersions are considered as not electronically conductive since their conductivity is about 0.01 mS/cm, or below. We note that it is difficult to measure electronic conductivities lower than 0.01 mS/cm with our method.

This is due to a residual ionic conductivity that is impossible to avoid, even after a long stabilization of the current. This residual conductivity may involve faradaic processes at the surface of the electrodes as described in supplemantal information.

Nevertheless, there is a substantial increase in electronic conductivity with increasing the concentration of particles. As shown in Figure 2, the conductivity curve displays an s-shape with a percolation threshold around 5.5 wt%. A conductive network is formed at this threshold which allows the suspension to be considered as electronically conductive. Electrical percolation is generally associated to rheological percolation with a large increase in viscosity (Barrie et al., 2004; Richards et al., 2017; Aoki and Watanabe, 2004; De Gennes, 1976; O'Mahony et al., 2019).

Nevertheless, the percolation behavior and the steepness of the curves depend on finite size effects (Saberi, 2015). The finite size of the sample is relative to other characteristic lengths including the size of the particles, and the minimal distance the particle clusters have to be within, so that they can be considered as electrically or elastically connected. In addition, these dimensions can display polydispersity which also affects the percolation behavior (Meyer et al., 2015). Here, we see that the conductivity increases more sharply with concentration than the viscosity.

The increase of conductivity is also sharper than the increase of yield stress (see Figure S3B supplemental information). Those differences result from distinct physical mechanisms. Electrical connectivity is related to the ability of electrons to be transported from one cluster to another cluster, whereas rheological connectivity is related to hydrodynamic interactions and to the ability to transfer elastic stress. Even if details of the particle interactions and of configurations of adsorbed polymers at their interface are not known, it can be expected that electrical transport and rheological behavior involve mechanisms with distinct length scales. Therefore, the shape of the percolation curves can appear as different. But globally they reflect the formation of networks that can both sustain elastic stress and transport electrical charges with increasing the concentration of carbon black particles.

However, the present dispersions at 7.0 wt% remain fluid enough to easily flow in our test setup without showing any phenomena of occlusion or clogging. For this reason, it was chosen as a specific concentration of carbon dispersions able to act as a percolated carbon flowable electrode. More importantly, and as shown later, the suspensions remain electrically conductive under flow, meaning that the conductive network is not disrupted by the shear.

The improvements of conductivity compared to previously investigated related materials can be ascribed to the use of large amounts of ammonium sulfate in the present work. Ammonium sulfate contributes to screening electrostatic repulsions between the carbon black particles. As a result, better electrical contacts can form between the particles. These improvements of electrical contacts result in a greater conductivity of the suspension.

### Electronic conductivity under shear

**Figure 3:**
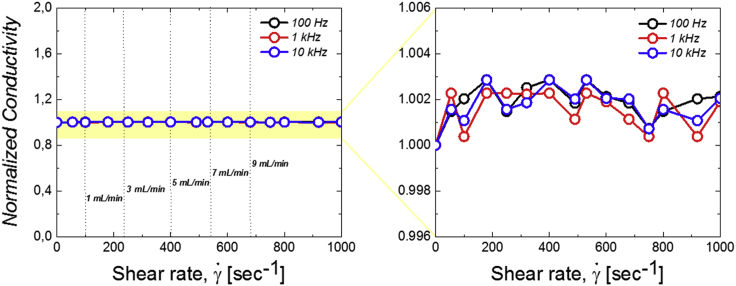
Electro-rheological behavior of a Ketjenblack 7.0 wt% dispersion in arabic gum 1.5 wt% – sodium alginate 0.5 wt% in (NH_4_)_2_SO_4_ 2 M The values of the normalized electronic conductivity are shown at the frequency of 100 Hz (black line), 1 kHz (red line), and 10 kHz (blue line). The data are normalized by the static reference value at 0 shear rate.

AC electrical measurements under shear have been performed using the setup described in supplemental information. Figure 3 shows the effect of the shear rate on the electrical conductivity of the sample under study. Because of experimental limitations, we cannot investigated shear rates above 1000 s ^−1^. Nevertheless, the investigated range of shear rates covers the shear rates in the electrochemical experiments described further [see supplemental information].

Unlike other studies in which the electrical properties under shear flow often reveal a breaking-and-reforming mechanism of aggregates (Helal et al., 2016; Hatzell et al., 2015; Narayanan et al., 2017) our system does not show any significant variation of the electrical properties under shear. These results suggest that the flow does not strongly alter the structure of the electrical network. It is also interesting to note that this behavior is somehow consistent with the rheological properties previously mentioned. Indeed, the present carbon black suspensions clearly display a shear thinning behavior. But the exponent associated to this behavior in the Herschel-Bulkley model remains quite high, of about 0.7. This value suggests a weak coupling between network structure and flow.

### Electrochemical characterization of flowable electrodes

For the electrochemical characterizations, the flowable carbon dispersions were tested in a two-electrode symmetric cell configuration. The carbon flowable electrodes have the same volume. They are separated from each other by using an anion exchange membrane (SnakeSkinDialysis Tubing 1000 MWCO).

**Figure 4:**
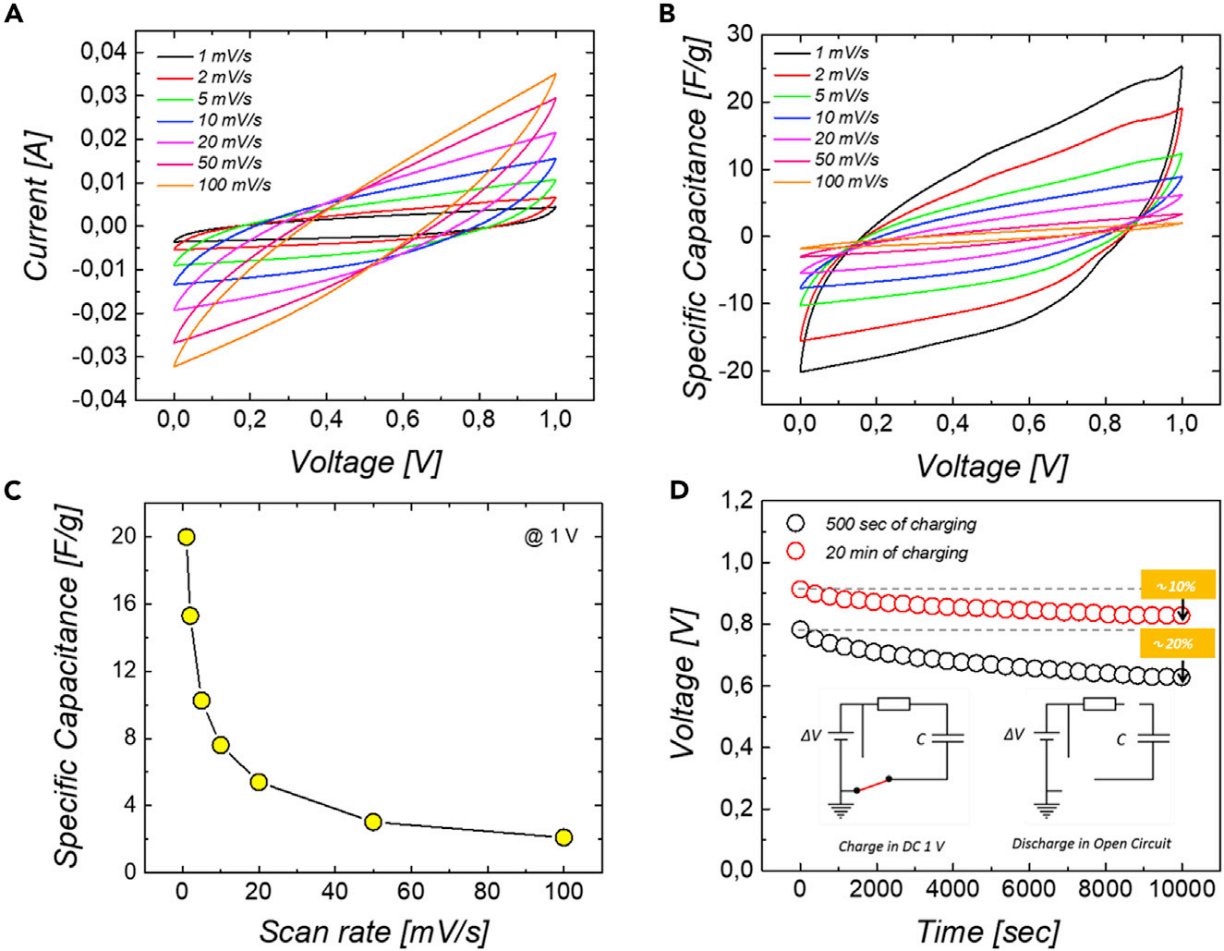
Electrochemical characterization of Ketjenblack aqueous dispersion as flowable electrode (A and B) (A) Cyclic voltammograms and (B) relative specific gravimetric capacitance of Ketjenblack dispersion 7.0 wt% in Arabic Gum 1.5 wt% and Sodium Alginate 0.5 wt% in (NH4)_2_SO_4_ 2 M. (C) Specific gravimetric capacitance against scan rate at 1mV/s. (D) Self-discharge measurements in OCV (Open Circuit Voltage) conditions.

Figure 4A shows the cyclic voltammetry (CV) curves of the carbon dispersions at different scan rates. A large capacitive current is observed due to the charging of the particles. Nevertheless, the electrical behavior of the sample does not show a typical rectangular shape characteristic of the charging of a capacitor formed by the adsorbed ionic double layers. The distortion of the CV curves suggests that there is a large resistive contribution in the system. The resistances are due to different factors, such as the intimate contact of the dispersions with the current collectors for the injection and collection of charges, and the contact resistances between the particles (Hatzell et al., 2017). Figure 4B shows the specific gravimetric capacitance *C*_*sp*_ of the sample calculated using the equation:

(Equation 2) $$C_{sp}=\frac{2i}{m\cdot \left(\frac{dV}{dt}\right)}$$

where, *i* is the value of measured current in the CV curves and *dV*/*dt* is the scan rate (Presser et al., 2012). *C*_*sp*_ is found to be rate-dependent, with the highest values obtained at low scan rate.

In particular, a specific capacitance of 20 F/g is calculated at 1 mV/s as shown in Figure 4C. The influence of scan rate presumably originates from the specie diffusion kinetics related to the porosity of the material with a broad range of characteristic sizes. The smallest pores are less accessible than bigger ones. At fast scan rates, it is likely that the small pores do not contribute to the storage of charges, resulting in a lower effective capacitance.

It is observed that the materials maintain their charges over a long time after charging. As shown in Figure 4D, the voltage across a cell containing 5 g of materials cell in an open-circuit configuration (Andreas, 2015; Chen et al., 2014; Niu et al., 2004; Ike et al., 2016; Xia et al., 2018) decreases by less than 10% after 10,000 s for a suspension that has been charged at 1V during 20 min.

The self-discharge is of about 20% for a suspension charged during 5 min at the same voltage. The self-discharge arises from losses in the circuit in which an oscilloscope is implemented, from redistribution of ions at the surface of the particles and from possible uncontrolled redox reactions with impurities.

### Flow-assisted charge and discharge

**Figure 5:**
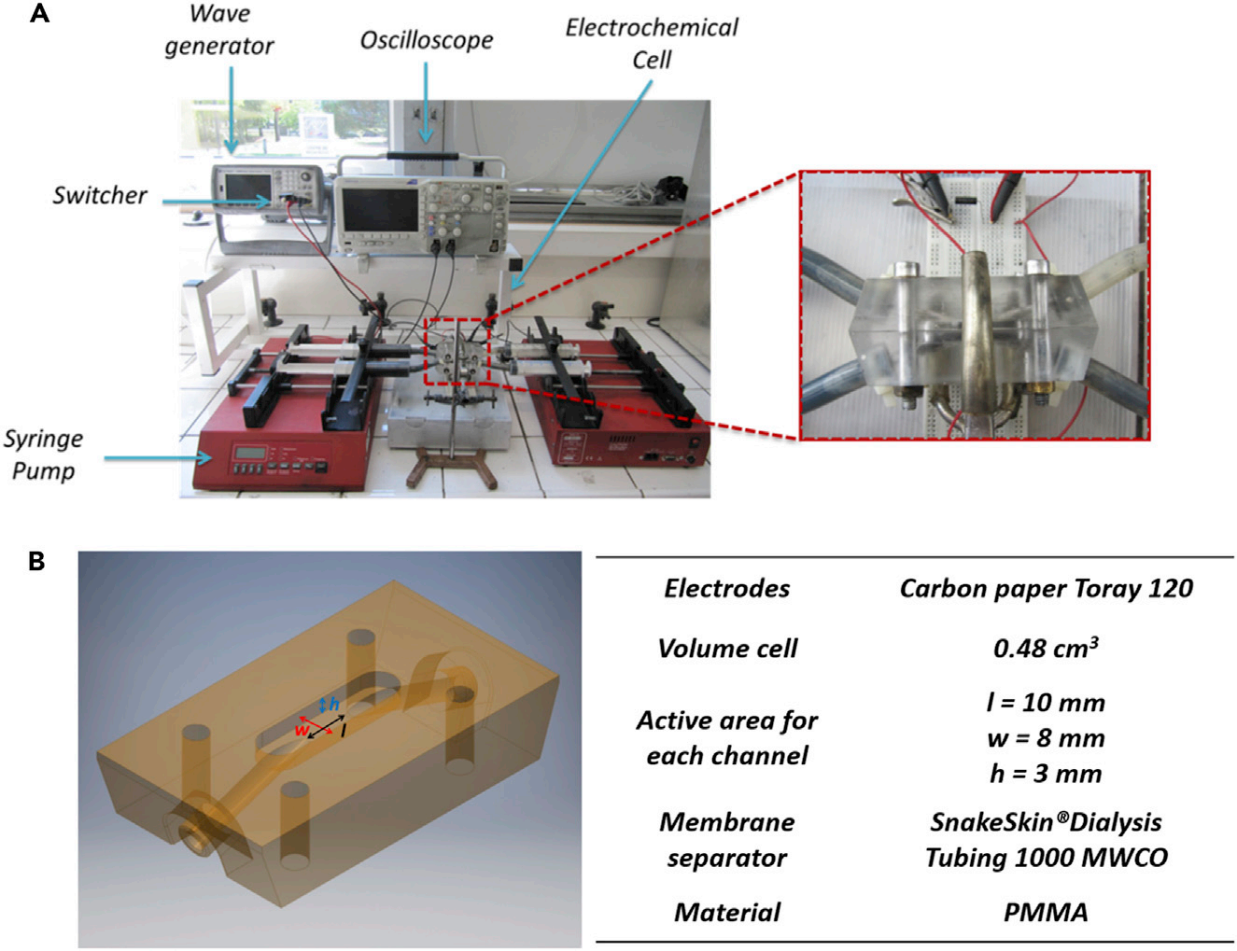
Experimental setup for flow-assisted operations (A) Experimental setup for flow-assisted charge and discharge. (B) Geometry and materials used in the electrochemical two-compartment cell.

In order to verify the ability of these flowable electrodes to transport and store charges, flow-assisted charge and discharge processes have been performed. During the charging process, the introduction of the new uncharged dispersion into the electrochemical cell, leads to a leak of current and to a decrease of the voltage across the cell below the values observed at rest. Conversely, during the discharge process, the reintroduction of charged carbon dispersions in the active area of the electrochemical cell leads to an increase of the voltage across the cell above the values observed at rest. This behavior is investigated by using the home-made experimental setup shown in Figure 5. The two-compartments electrochemical cell was connected by means of silicone tubes to 4 syringes. The latter act as the reservoirs of the charged and uncharged dispersions. This electrochemical cell is connected to an RC circuit which is directly connected to a wave-form voltage generator (Trueform 33500B, Keysight). The charging process is performed at 1 V for 200 s by using a load resistance of 178 Ω. In this phase, the uncharged carbon dispersion is pumped by syringe pumps through the electrochemical cell, where electric charges are supplied to the dispersions.

**Figure 6:**
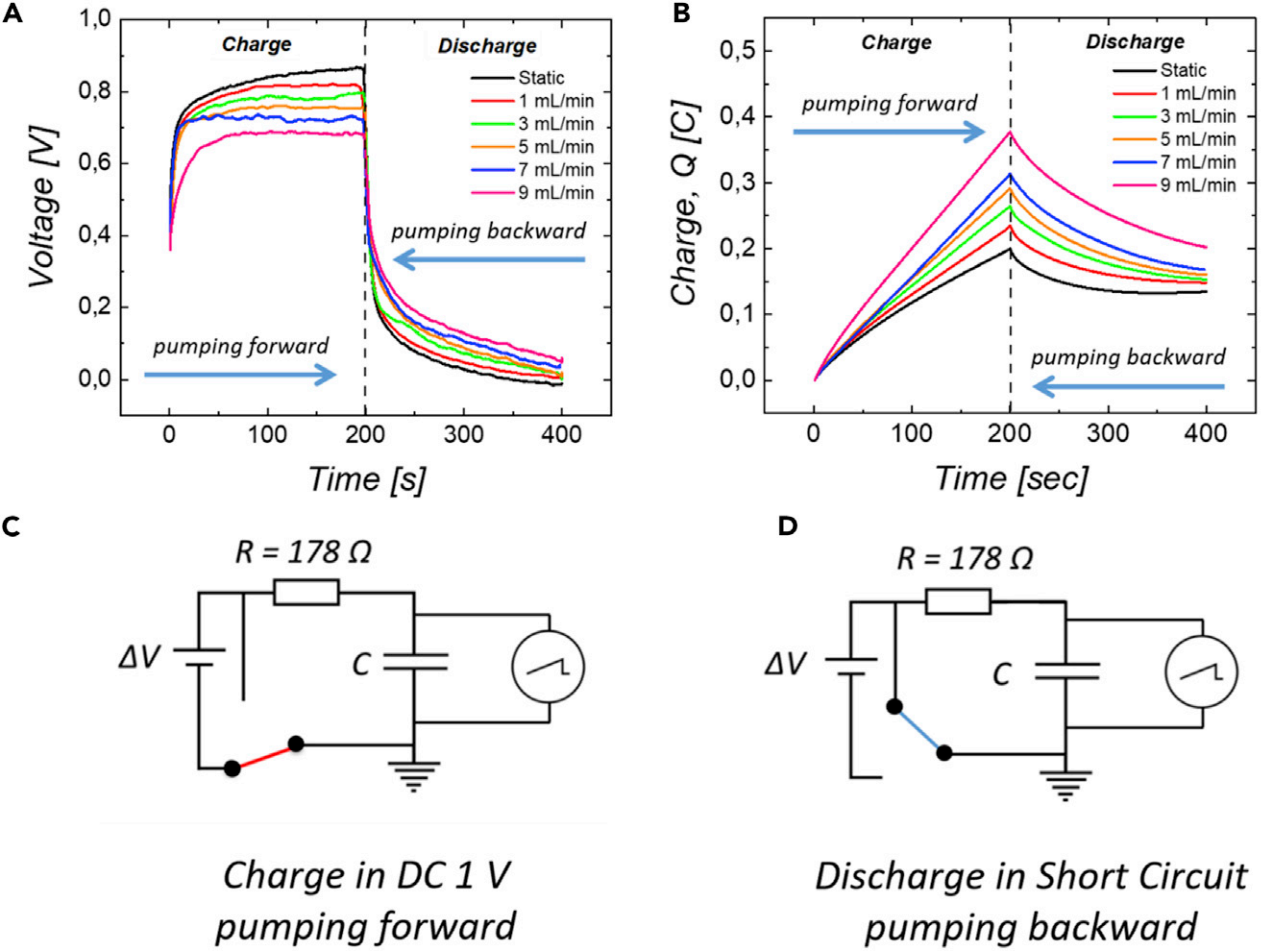
Flow-assisted operations (A) Charge and discharge processes of flowable carbon electrodes. (B) Stored and converted charge profiles during the charge-discharge processes at different flow rates. (C) Scheme of the electrochemical circuit during the charging process in DC at 1 V. (D) Scheme of the electrochemical circuit during the discharging process in short circuit conditions.

The charged carbon dispersions are collected in the other two syringes. During the discharge process, the circuit is short-circuited by means of a switch. The voltage across the electrochemical cell, was recorded by an oscilloscope (Tektronix DPO, 2022B). Figure 6A show the charge and discharge processes of the formulated flowable carbon electrodes. These processes are characterized by two specific times, *t*_1_ and *t*_2_. During the charging process, the voltage across the cell reaches its maximum value at *t*_1_ = 200 *s*. This initial process is followed by a second one of discharge, of the same duration, in which the voltage across the cell reach its minimum at *t*_2_ = 400 *s*.

The black curve represents the profile of charge and discharge of the dispersion in static conditions. As shown in Figure 6A, at *t*_1_ = 200 *s* the voltage across the cell reaches the value of 0.86 V and approaches zero after discharge at *t*_2_ = 400 *s*. At a flow rate of 1 mL/min (red curve), the effect of flow keeps the voltage across the cell constant at a lower value than a static one. At *t*_1_ = 200 *s* the process of discharge of the particles begins. During this process in dynamic conditions, the voltage reaches a value greater than that under static conditions.

Similar trends are found for the other flow rates up to 9 mL/min. Table 3 shows the final charge and discharge values of the flowable electrodes. These results represent a proof of concept of the use of flowable electrodes to store and transport charges. Note that in the investigated range of fluxes, from 0 to 9mL/min, and as previously show, the conductivity of the suspension is not expected to vary. Indeed, in this range, the shear rates remain below 1000 s ^−1^ [see supplemental information].

**Table 3:** Voltage across the cell under charge and discharge at time *t*_1_ and *t*_2_. The materials are charged from 0 s to *t*_1_and discharged from *t*_1_ to *t*_2_

Time	Static	1 mL/min	3 mL/min	5 mL/min	7 mL/min	9 mL/min
*t*_1_ = 200 *s*	0.86 V	0.81 V	0.78 V	0.74	0.72	0.67
*t*_2_ = 400 *s*	0 V	0.005 V	0.014 V	0.018 V	0.035 V	0.053 V

From such charge-discharge measurements, it is possible to calculate the stored charges within the system and to analyze the effect of the flow using Equation 3.

(Equation 3) $$\int \frac{V_{in}-V_{out}}{R}\;dt=\;\int \frac{V_{R}}{R}\;dt=\;\int idt=Q$$

Where *V*_*in*_ stands for the potential applied to the circuit, and *V*_*out*_ for the potential across the electrochemical cell at time *t.* The stored charge evolution during the charge and discharge processes of the carbon flowable electrodes under flow is shown in Figure 6B. At time *t*_1_ = 200 *s* the amount of charge is equal to 0.19 C in static conditions. The effect of flow results in an increase of stored charges during the charging process but also of restored charges during discharge. Charge values during the two processes are given in Table 4. From these considerations, it is possible to carry out a deeper analysis of the effect of flow on the charge and discharge processes of the carbon flowable electrodes.

**Table 4:** Stored and restored charges in C during the processes of charge and discharge at time *t*_1_ and *t*_2_. Charges stored up to *t*_1_, restored charges from *t*_1_ to *t*_2_

	Static	1 mL/min	3 mL/min	5 mL/min	7 mL/min	9 mL/min
*t*_1_ = 200 *s*	0.19 C	0.23 C	0.26 C	0.28 C	0.31 C	0.37 C
*t*_2_ = 400 *s*	0.13 C	0.14 C	0.15 C	0.16 C	0.17 C	0.20 C

**Figure 7:**
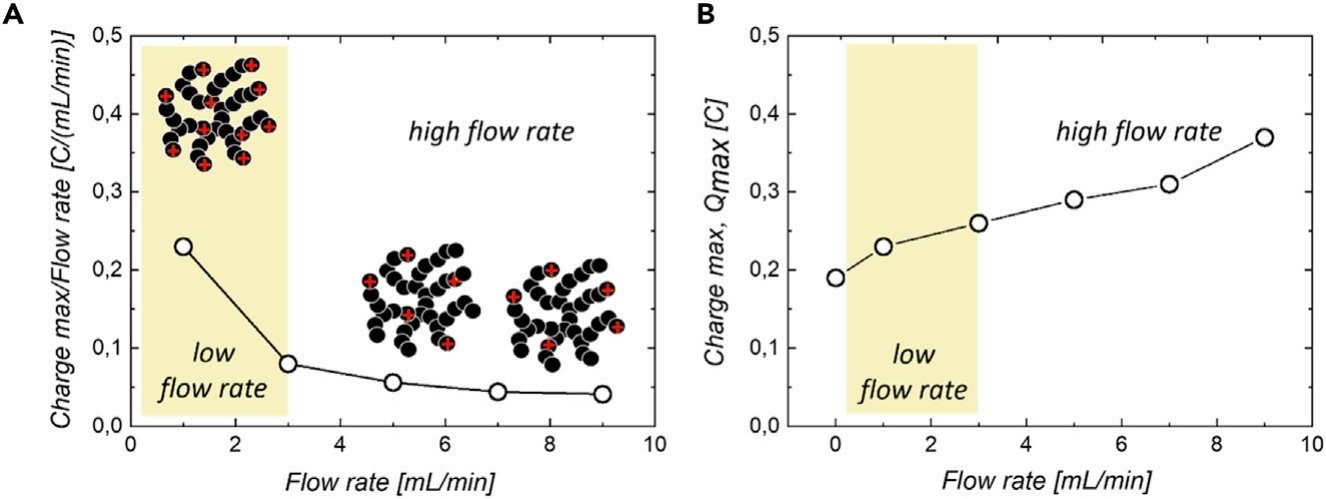
Effect of the flow on the stored charge in dynamic conditions (A) Normalized maximum charge stored at time t_1_ = 200 s against the flow rate. More clusters are involved in the transport of charges at high flow rate, but the charge per clusters is lower than that at low flow rate. As a net result, the total amount of charges is still greater at high flow rate. (B) Maximum charge acquired by the system as function of the flow rate.

Figure 7A shows the evolution of the normalized maximum charge stored at time *t*_1_ against the flow rate. Normalization by the flow rate allows the efficiency of the charging process to be evaluated. Indeed, for a similar efficiency, one would expect an amount of stored charges proportional to the flow rate, considering that the amount of charges is directly proportional to the amount of materials transported through the cell. Here, it is observed that the efficiency is greater at low flow rate, meaning that the amount of charges per carbon particle decreases with the flow rate. Nevertheless, in spite of a lower efficiency, the net amount of stored charges still increases with the flow rate. The system still transports a greater amount of charges at high flow rate, even if fewer charges are transported at the level of each carbon particle. The value of maximum charge acquired by the system as function of the flow rate is shown in Figure 7B.

Last, it is also interesting to estimate the power supplied during discharge through the load resistance. This power is given in Figure S5 for a load resistance of 178 Ω and at time *t*_*2*_ of 400 s. The power remains rather low but increases strongly with the flow rate. It reaches a value of 0.02 mW for a flow rate of 9 mL/min.

### Conclusion

The overall objective of this work focuses on the study and implementation of a highly conductive colloidal carbon based suspension as flowable electrode for FAESs. We have used an aqueous dispersion of carbon materials in the presence of arabic gum as surfactant and sodium alginate as stabilizer. We have shown that the addition of ammonium sulfate at high concentration allows a substantial improvement of properties compared to previous studies. In order to improve the rheological and electrical performances of these carbon dispersions, a different formulation protocol was adopted. The materials presented in this work display a high electronic conductivity of 65 mS/cm, two orders of magnitude higher than related flowable carbon dispersions proposed in the literature. The formulated dispersion shows a high viscosity but still lower than most values reported in the literature. The specific gravimetric capacitance of 20 F/g is not high, but the materials have been shown to be efficient to store and restore charges in flow conditions. Overall, the progresses made in terms of the conductivity and viscosity allow for a faster charging of the electrodes and easier circulation with less energy lost in pumping the fluids. Future work should be focused on improving the capacitance, using for example more porous carbon black, to make the present formulations still more efficient for future energy management technologies.

### Limitations of the study

The main limitation of the study is the technical limitation of shear rates investigated. As discussed in the main text and in supplemental information the present results and conclusions are obtained for shear rates up to 1000 s^−1^.

### Resource availability

#### Lead contact

Further information and requests for resources should be directed to and will be fulfilled by the lead contact, Philippe POULIN (philippe.poulin@crpp.cnrs.fr)

#### Materials availability

This study did not generate new unique reagents.

#### Data and code availability

The work does not include any unpublished custom code, software, or algorithm that is central to supporting the main claims of the paper. The work does not contain any particular type of biological data.

## Methods

All methods can be found in the accompanying transparent methods supplemental file.

## Footnotes

Supplemental information can be found online at https://doi.org/10.1016/j.isci.2021.102456.
