# Supplementary material for: Highly conductive colloidal carbon based suspension for flow-assisted electrochemical systems
Source: iScience. 2021 Apr 20;24(5):102456. doi: 10.1016/j.isci.2021.102456 (PMC8113993; doi:10.1016/j.isci.2021.102456)
Supplement: Document S1. Figures S1–S7 [file mmc1.pdf]

iScience, Volume *24*

Supplemental information

Highly conductive colloidal carbon based suspension for flow-assisted electrochemical systems

Marco S. Alfonso, Hélène Parant, Jinkai Yuan, Wilfrid Neri, Eric Laurichesse, Katerina Kampioti, Annie Colin, and Philippe Poulin

Supplemental Information

Materials and methods

Sample preparations

Carbon black dispersions are prepared by mixing the carbon particles (Ketjenblack EC 600JD AkzoNobel) within an aqueous solution. The latter contains 1.5 wt% of arabic gum (Molekula, CAS Number 9000-01-5), 0.5 wt% sodium alginate (Aldrich, CAS Number 9005-38-3), and 2 M ammonium sulfate (Aldrich, CAS Number 7783-20-2). Arabic gum acts as a highly effective surfactant for the dispersion of carbon particles whereas sodium alginate serves as stabilizer against sedimentation. The used stabilizers ensure a constant conductivity of the dispersion over time. The solution was heated for 1 h at 80 °C under magnetic stirring until complete dissolution of polymers.

Subsequently, the grinding of the carbon powder was processed with the aid of a mortar in order to obtain a fine control of the size of the particles. This step is followed by a heat treatment of the powders in the oven at 180 °C for 2 h. This procedure facilitates the wettability of the carbon particles and consequently their dispersibility. After cooling, carbon black particles were added to the aqueous solution with the desired proportions, and the blend is kept under stirring for several minutes until the carbon black is totally wetted by the aqueous solution.

Finally, to homogenize the dispersion, mechanical shear stress was applied with a high shear mixer (Silverson L4RT) for 30 min at a given rotation speed of 5000 rpm, as shown in **Figure S1a**, corresponding to an applied shear rate of 3500 sec^{-1}. The obtained colloidal dispersion is shown in **Figure S1b**.

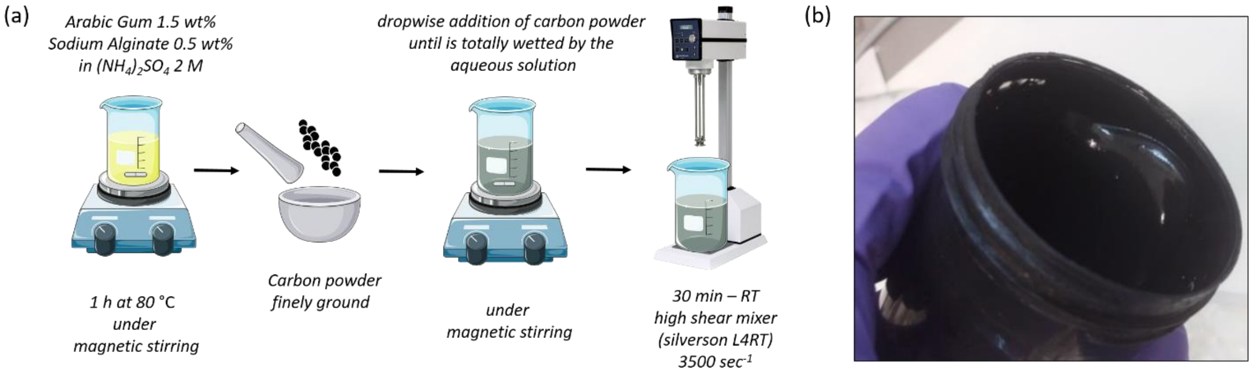

Figure S1 – Scheme of the formulation protocol of colloidal carbon flowable electrodes.(a) Scheme of the formulation protocol. (b) Colloidal carbon based flowable electrodes at 7.0 wt%, Related to Figure 1.

Microstructure Morphologies

Ketjenblack dispersions samples were deposited between glass slides for optical imaging by using a Leica DM 2500P microscope. Below, the optical micrographs of some dispersions at different concentrations of carbon black.

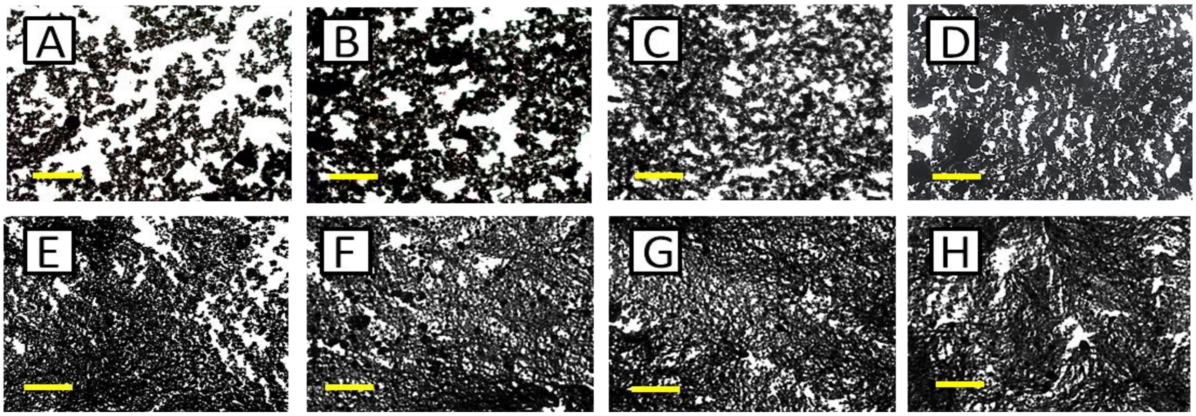

Figure S2 – Optical micrographs of Ketjenblack dispersions in arabic gum 1.5 wt% - sodium alginate 0.5 wt% in $(NH_4)_2SO_4$ 2 M. (A) 2.0 wt%, (B) 3.0 wt%, (C) 4.0 wt%, (D) 5.0 wt% (E) 6.0 wt%, (F) 7.0 wt%, (G) 8.0 wt%, (H) 9.0 wt% (Scale bar 50 μm), Related to Figure 1

Rheology characterizations

The viscosity and the shear stress were measured with an AR1000 controlled stress rheometer from TA instrument. The geometry used is a PMMA disc-plate of 60 mm in diameter, with a gap of 800 μm relative to the bottom plate as shown in **Figure S3a**.

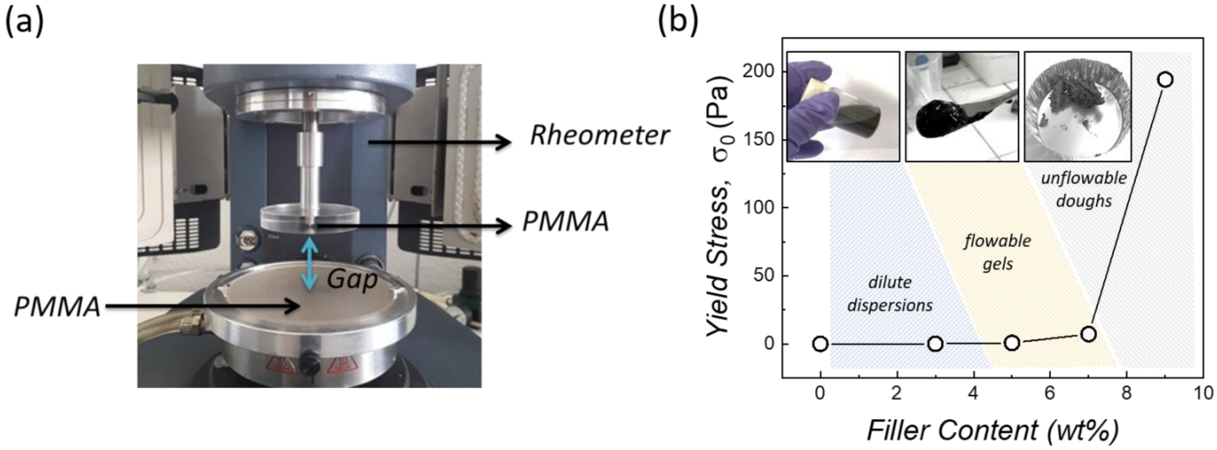

Figure S3 – Rheology characterizations of colloidal carbon flowable electrodes. (a) Rheometer (TA instrument) set-up for viscosity and shear stress measurements. (b) Yield stress values obtained by using the Herschel-Bulkley law of the Ketjenblack aqueous dispersion composed of 1.5 wt% arabic gum, 0.5 wt% sodium alginate and 2 M $(NH4)_2SO_4$. (Inset: Photos showing three different concentrations of Ketjenblack dispersions: dilute dispersion, flowable gel and unflowable dough), Related to Figure 1.

Several tests were conducted on the choice of the gap to demonstrate that the results are independent on the width gaps larger than 500 µm. A ramp of shear rate is applied from 500 sec^{-1} to 0.1 sec^{-1}, with 10 points per decade. Each shear rate is maintained during 30 seconds and the viscosity is measured by averaging the measurements over the last 5 seconds. The temperature is set with a Peltier system at 20° C.
The formulated carbon dispersions exhibit a flowable behavior with low values of yield stress up to the concentration of 7.0 wt% of active material. Above this concentration, the dispersion turns into unflowable dough in which the yield stress rises sharply as shown in **Figure S3b.**

Electronic conductivity measurement

In order to determine the electrical percolation threshold, DC chronoamperometry measurements have been performed at different concentrations of carbon in the dispersion. The conductivity involves ionic and electronic contributions.
Ionic conductivity is not negligible due to the presence of charged species from the dissolved polymers (sodium alginate and arabic gum) and ammonium sulfate. But the electronic conductivity can be determined at "zero frequency" condition. To this end, the current is measured after a certain time in order to eliminate the contribution of current involved in the polarization of the electrodes by the free ions in solution.
The measurements are carried out with a two-plate capacitor cell connected to a potentiostat/galvanostat (Metrohm Autolab/PGSTAT101). The platinum electrodes of this cell are symmetrical with a square surface A= 3 mm x 3 mm and separated by a distance d=3 mm. The cell probe is immersed in the carbon dispersion over a height of 1.5 cm. A DC voltage of ± 1 V is applied to the electrodes and the current is measured for a long period of time (2000 sec) until the polarization current decreases to zero as shown in the manuscript in **Figure 2**.
The stabilized current $I_{plateau}$ allows us to measure the electrical resistance between the two electrodes, and the electronic conductivity knowing the cell geometry:

$$\sigma_e = \frac{I_{plateau}}{|E|} \cdot \frac{d}{A} \qquad \text{(equation S1)}$$

where $I_{plateau}$ is the stabilized current measured at 2000 sec, $|E|$ is the applied voltage, d is the distance between the electrodes and A is the surface area of the electrodes.

AC electrical measurements under shear

The electrical impedance measurements under shear were performed by using a Couette cell (Caplim Rheophysique West 3400) connected to the impedancemeter as shown in **Figure S4a.**
This experimental set-up enables the analyzis of the dynamic properties of the sample in a wide range of shear rates up to 1000 sec^{-1}. This apparatus consist of a specific cylindrical sample-holder in Polyether ether ketone (PEEK) using blocking circular gold electrodes with surface areas of 770 mm^2, resistance of

0.3 Ω and thickness of 0.6 mm. In order to obtain the apparent shear rate values from the Couette cell revolutions, a conversion protocol was carried out. Through the speed of revolution ω, the apparent shear rate $\dot{\gamma}$ is deduced using the following equation:

$$F_{\dot{\gamma}} = \frac{R_2^2 + R_1^2}{R_2^2 - R_1^2} \quad \text{(equation S2)}$$

where the apparent shear rate $\dot{\gamma}$ is :

$$\dot{\gamma} = F_{\dot{\gamma}}\omega \quad \text{(equation S3)}$$

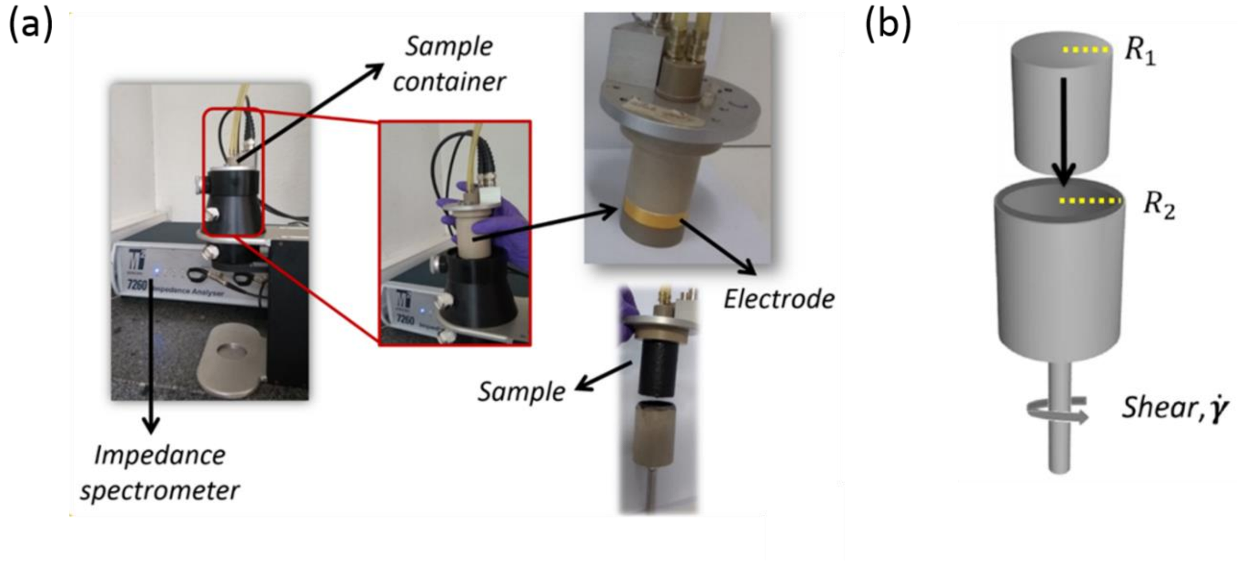

Figure S4 – AC electrical measurements under shear. (a) Experimental setup for the AC electrical measurements under shear. (b) Schematic representation of the sample-holder geometry in AC electrical measurements under shear ($R1$ = 0.01691 m, $R2$ = 0.01751 m and $F\dot{\gamma}$ = 28.69), Related to Figure 3.

Power during discharge

The power supplied in a load resistance *R* of 178 Ω is calculated as RI^2, where *I* is the discharge current. The obtained values are shown in **Figure S5.**

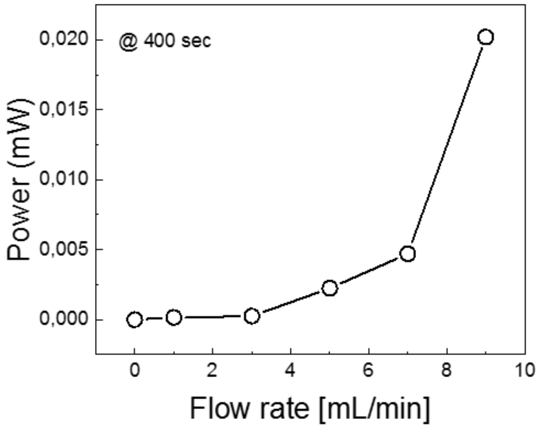

Figure S5 – Power during discharge. Power supplied through a load resistance of 178 Ω at time t_2=400 sec as function of flow rate, Related to Figure 6.

Estimate of shear rate in the electrochemical flow cell

Since the flow rate is geometry-dependent, the most useful parameter for these studies is the shear stress at the wall of the channel. The conversion from flow rate to shear stress is done by using the model of the laminar flow inside two-plates channel.

This model foresees a uni-directional flow rate showing variations of the velocity gradient along the axis ($\vec{z}$) due to the shear stress effects at the boundaries.

These effects involve a variation of the velocity gradient, as shown in **Figure S6**, with a decrease of it at the walls (Areas I) and a flow rate with constant velocity gradient in the center of channel (Area II).

The rectangular geometry of two-compartments electrochemical cell, used in the flow dependent measurements, assures the aforementioned laminar flow rate features.

In details the width of the channel of the electrochemical cell (w = 0.8 cm) is larger than its thickness (h = 0.3 cm) for compartment, and it has a length of (l = 1 cm).

Under these assumptions, the drop of pressure $\Delta Pz = P_{in} - P_{out} > 0$ between the inlet and the outlet, the shear stress $\sigma_{(z)}$, the shear rate $\dot{\gamma}_{(z)}$ and the velocity gradient $v_{(z)}$ are linked by the following relations:

$$\sigma_{(z)} = \frac{\Delta P_z}{l} \quad \text{and} \quad \dot{\gamma}_{(z)} = \frac{dv(z)}{dz} \qquad \text{(equation S4)}$$

where, *z* is the coordinate along the height direction ($\vec{z}$), and it is equal to zero in the middle of the channel.

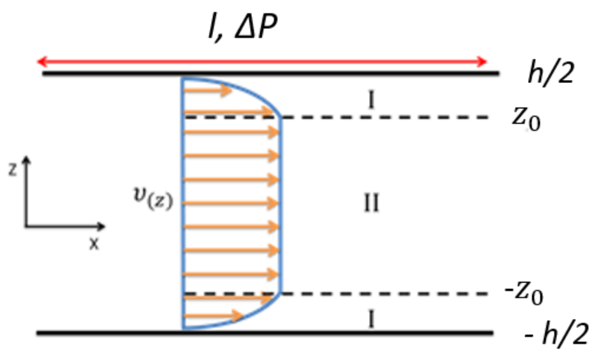

Figure S6 - Schematic diagram pressure-driven for laminar flow. The flow is uni-directional in the direction of the pressure gradient. Area (I) represents the shear stress effects at the boundaries. Area (II) represents the top-front of the fluid with constant velocity, Related to Figure 6.

To calculate the velocity gradient, we fit the flow curve that correlates the shear rate $\dot{\gamma}_{(z)}$ and the shear stress $\sigma_{(z)}$ with the Herschel-Bulkley equation:

$$\sigma_{(z)} = \sigma_0 + k\dot{\gamma}\,(z)^n \qquad \text{(equation S5)}$$

that can be expressed through the formula S1, obtaining:

$$\frac{\Delta P_z}{l} = \sigma_0 + k\left(\frac{dv}{dz}\right)^n \qquad \text{(equation S6)}$$

where, σ_0 is the yield stress and k and n are parameters deduced from the fitting of the experimental rheological curves reported in Table 2.

The rheological parameters used for this conversion are those obtained for a dispersion of Ketjenblack 7.0 wt% in arabic gum 1.5 wt% - sodium alginate 0.5 wt% in $(NH_4)_2SO_4$ 2 M used in this work.

After calculations, we obtained the velocity gradient profile $\vec{v}(z)$. This gradient is valid for $z \geq 0$, but for $z \leq 0$, it is necessary to change the sign of z because the profile is symmetric with respect to $z = 0$. Therefore, by rearranging the equation (S6) we obtained:

$$\frac{dv(z)}{dz} = \frac{lkn}{(n+1)\Delta P}\left(\frac{\frac{\Delta P_z}{l} - \sigma_0}{k}\right)^{\frac{1}{n}} \qquad \text{(equation S7)}$$

which after integration was expressed by the form:

$$v_{(z)} = \frac{lkn}{(n+1)\Delta P}\left(\frac{\frac{\Delta P_z}{2l} - \sigma_0}{k}\right)^{\frac{n+1}{n}} + constant \qquad \text{(equation S8)}$$

The previous expression can only be solved if $\frac{\Delta P_z}{2l} - \sigma_0 \geq 0$, where $z \geq \frac{l\sigma_0}{\Delta P}$.

The value $z = \frac{l\sigma_0}{\Delta P}$, represents the limit between the area near the wall of the channel, where the shear stress has a higher impact and the area of the center of the channel at constant velocity. Given the roughness of the carbon paper electrodes (around 20 µm), we can safely assume a no-slip condition at the boundaries, $v\left({}^{h}/_{2}\right) = 0$, obtaining:

$$v_{(z)} = \frac{lkn}{(n+1)\Delta P}\left[\left(\frac{\frac{\Delta Ph}{2l} - \sigma_0}{k}\right)^{\frac{n+1}{n}} - \left(\frac{\frac{\Delta P_z}{2l} - \sigma_0}{k}\right)^{\frac{n+1}{n}}\right] \qquad \text{(equation S9)}$$

The same calculation for the negative *z* gives the same velocity profile $v_{(X)}\vec{h}_z$ in the channel. These equations describes the flow of the carbon dispersions close to the walls of the channel $(\text{for } z \geq \frac{l\sigma_0}{\Delta P})$, characterized by a high shear-stress.

$$\vec{v}(z) = \begin{cases} \frac{lkn}{(n+1)\Delta P}\left[\left(\frac{\frac{\Delta Ph}{2l} - \sigma_0}{k}\right)^{\frac{n+1}{n}} - \left(\frac{\frac{\Delta P_z}{l} - \sigma_0}{k}\right)^{\frac{n+1}{n}}\right] & for \quad \frac{l\sigma_0}{\Delta P} \leq z \leq \frac{h}{2} \\ \frac{lkn}{(n+1)\Delta P}\left(\frac{\frac{\Delta Ph}{2l} - \sigma_0}{k}\right)^{\frac{n+1}{n}} & for \; -\frac{l\sigma_0}{\Delta P} \leq z \leq \frac{l\sigma_0}{\Delta P} \\ \frac{lkn}{(n+1)\Delta P}\left[\left(\frac{\frac{\Delta Ph}{2l} - \sigma_0}{k}\right)^{\frac{n+1}{n}} - \left(\frac{\frac{-\Delta P_z}{l} - \sigma_0}{k}\right)^{\frac{n+1}{n}}\right] & for \; -\frac{h}{2} \leq z \leq -\frac{l\sigma_0}{\Delta P} \end{cases}$$

After integration of $\vec{v}(z)$, a relationship between the flow rate *Q* and the pressure difference *ΔP* was found as follow:

$$Q = \frac{hlknw}{(n+1)\Delta P}\left(\frac{\frac{\Delta Ph}{2l} - \sigma_0}{k}\right)^{\frac{n+1}{n}} - \frac{2w(lkn)^2}{(2n+1)(n+1)\Delta P^2}\left(\frac{\frac{\Delta Ph}{2l} - \sigma_0}{k}\right)^{\frac{n+1}{n}} \qquad \text{(equation S10)}$$

By using this relationship, the flow rate Q in (mL/min) can be finally converted into pressure difference ΔP (Pa). Then, the shear stress at the wall and the corresponding shear-rate for a given dispersion is found at $z = \pm {}^{h}/_{2}$ replacing them in expression S1:

$$\sigma_w = \frac{\Delta P h}{2l} \qquad and \qquad \dot{\gamma} = \left(\frac{\sigma_w - \sigma_0}{kn}\right)^{\frac{1}{n}}$$

Through this procedure, it is possible to calculate the shear stress and the corresponding shear rate of a sample with known viscosity for a given geometry. In **Figure S7a** is reported the correlation between the flow-rate and correspondent shear stress at wall.

Subsequently the obtained flow dependent shear rate for the specific geometry and known rheological behavior is shown in **Figure S7b**.

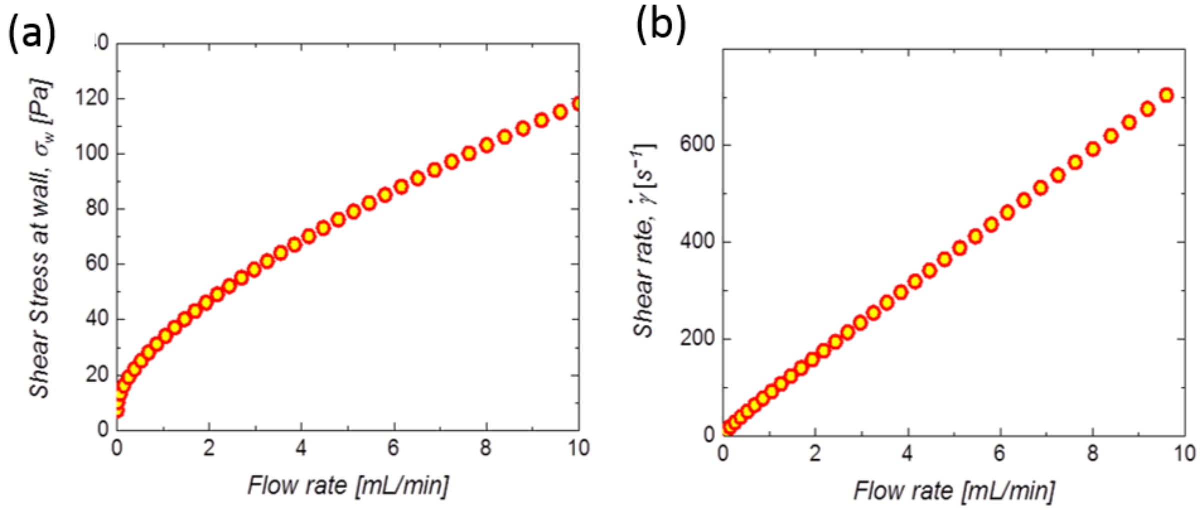

Figure S7 – Estimate of shear rate in the electrochemical flow cell. (a) Correlation between the flow-rate and correspondent shear stress at wall. (b) Shear rate obtained for the channel geometry (w=0.8 cm, h=0.3 cm and l=1 cm) for a specific carbon dispersion made of Ketjenblack 7.0 wt% in arabic gum 1.5 wt% - sodium alginate 0.5 wt% in $(NH_4)_2SO_4$ 2 M with (k= 1.25, σ_0 = 7.18, and n = 0.68), Related to Figure 6.
